# Fate of three bioluminescent pathogenic bacteria fed through a cascade of urine microbial fuel cells

**DOI:** 10.1007/s10295-019-02153-x

**Published:** 2019-02-22

**Authors:** Ioannis Ieropoulos, Oluwatosin Obata, Grzegorz Pasternak, John Greenman

**Affiliations:** 1 Bristol BioEnergy Centre, Bristol Robotics Laboratory, University of the West of England, Bristol, BS16 1QY UK; 2 Biological, Biomedical and Analytical Sciences, University of the West of England, Bristol, BS16 1QY UK; 3 Faculty of Chemistry Wroclaw, University of Science and Technology, Wyb. Wyspianskiego 27, 50-370 Wrocław, Poland

**Keywords:** Microbial fuel cells, Pathogen inactivation, Urine, MFC cascade, Terracotta

## Abstract

Microbial fuel cell (MFC) technology is currently gaining recognition as one of the most promising bioenergy technologies of the future. One aspect of this technology that has received little attention is the disinfection of effluents and the fate of pathogenic organisms that find their way into the waste stream. In this study, three independent trials were carried out to evaluate the fate of three bioluminescent pathogenic bacteria (*Salmonella enterica* serovar *Typhimurium, Pseudomonas aeruginosa* and *Staphylococcus aureus*) introduced into the anodic chamber of a urine-fed cascade of 9 MFCs with matured, electroactive biofilms. These are common examples of enteric human pathogens, which could contaminate urine or waste streams. The results showed that the average power generation in the closed circuit cascade reached 754 ± 16 µW, with an average pathogen log-fold reduction of 6.24 ± 0.63 compared to 2.01 ± 0.26 for the open circuit cascade for all three pathogens. The results suggest that the bio-electrochemical reactions associated with electricity generation were the primary driving force for the inactivation of the introduced pathogens. These findings show that pathogenic organisms introduced into waste streams could be inactivated by the power-generating process within the MFC cascade system, thereby preventing propagation and thus rendering the effluent safer for possible reuse.

**Electronic supplementary material:**

The online version of this article (10.1007/s10295-019-02153-x) contains supplementary material, which is available to authorized users.

## Introduction

The microbial fuel cell (MFC) is an innovative technology for the direct conversion of organic matter into electricity. MFCs exploit the unique ability of certain bacteria to donate electrons to an anode electrode, as part of their natural metabolism. Electricity is generated as a result of organic matter degradation as the anode becomes the terminal electron acceptor [6, 15, 26]. Interest in MFCs has grown significantly over the past decade possibly as a result of demonstrating a wide range of applications [32]. Although much of the interest in the technology has been fuelled by the quest for better wastewater management, due to more stringent water discharge limits [11], there are currently many other applications where MFCs could be implemented [6, 26]. More recently, the use of neat human urine as the sole feedstock has been widely reported with promising applications in powering portable devices such as mobile phones [12, 14, 23, 36]. Urine is particularly suited as a feedstock for MFC applications because of its abundance, composition of suitable nutrients for microbial growth, as well as its high conductivity [4, 23, 39, 41]. It has been demonstrated as a promising technology for addressing energy and environmental issues particularly in poor and remote areas of the world [1, 30].

Most of the current interest in MFCs is understandably focused on the optimisation of the overall power output by enhancing different aspects of the technology. Some of these include coating the carbon anodes with ionic liquid polymer [37], miniaturisation [14], controlling anodic biofilms [22] as well as the modification of other parameters involved in the MFC technology [3].

Although significant advances have been made in generally improving the performance of the MFC technology, other aspects have remained largely underexploited. One area of technological implementation, which has received very little attention, is the fate of pathogenic organisms that may be introduced into the system with potential public health implications resulting from its operation. Wastewater and urine treatment using MFCs carry the potential risk of faecal contamination and introduction of different associated pathogens into the waste stream. Improper waste management, resulting in faecal contamination of water and food resources, leads to thousands of deaths every year as a result of diseases caused by pathogenic organisms such as *E. coli* and *Salmonella* sp. [24]. This is still commonplace in sub-Saharan Africa [16], Southeast Asia [18] and South America [21], areas which would likely benefit the most from MFCs based on the favourable environmental conditions and the need for energy and sanitation [1]. It is therefore important to examine the fate of pathogenic organisms in waste streams during the MFC-led power generation process as well as post-MFC treatment, to evaluate the risk of infections. This is also of greater importance where the treated effluents are to be utilised in further applications such as irrigation.

Previous work has reported on the ability of MFCs or the MFC process by-products to suppress the growth of microorganisms. For example, a recent research in our lab conducted by Gajda et al. [8] highlighted the bactericidal properties of the synthesised catholyte from the cathodic chamber of ceramic-based MFCs treating wastewater. In their report, catholyte was applied to bioluminescent *E. coli* and monitored with a bench top luminometer with the results showing significant reduction (> 4 log-fold) in *E. coli* viability over a period of 120 s compared to the open circuit controls (< 1 log-fold over the same period). Although this previous report highlighted the unique application for the synthesised catholyte, it did not provide any information on antisepsis occurring in the anodic chamber.

Some unique characteristics of the MFC anodic chamber provide an indication for its ability to suppress the growth of exogenous microorganisms that might be introduced to it. As such, there is a possibility of exploiting these qualities to achieve disinfection of any contaminated influent—whether urine or wastewater. Some of these characteristics within the anodic chamber are hypothesised to be: (1) competition for energy source; (2) high pH (> 9.5) which can be obtained in urine-powered MFCs and the electroactive nature of power-generating MFCs.

Our recent study reported that the anodic chamber of ceramic-based, 3D-printed urine-powered MFCs could bring about considerable pathogen inactivation as a result of the power generation process [12]. In this previous research, real pathogenic *Salmonella enterica* serovar *Enteritidis* was introduced into the anodic chamber of the MFCs and monitored in real time in a cascade system. The results highlighted differences in disinfection efficacy between the power-generating cascade (closed circuit) and the open circuit cascade. The closed circuit cascade effected greater than four log-fold reduction in both viable counts and bioluminescence (of a bioluminescent genetically modified (GM) variant of the pathogen). The study indicated that pH, oxidation–reduction potential (ORP) and the electron flow achieved in the anodic chamber of closed circuit MFCs could suppress the growth and deactivate *S. enteritidis*, a diarrhoea-causing bacterium [12].

The current study reported herewith aims at investigating the fate of three different model pathogens introduced into two different microbial fuel cell cascade systems, with a well-developed electroactive biofilm community. These pathogens are often associated with faecal contamination and improper waste management, resulting in disease outbreaks [16, 18] and it is therefore important to assess their fate within the anodic chamber of urine-fed MFCs in a continuous flow system in situ. The choice of organism was mainly informed by the wide range of applications for MCF technology (such as in the treatment of domestic wastewater, faecal sludge including urine and hospital wastewater, amongst others). Although the tested organisms might not be directly obtained from neat urine, the possibility of faecal cross-contamination is high. Furthermore, *Pseudomonas aeruginosa* found in soil and water can cause urinary tract infections; as such, it is highly likely to be present in the urine of infected individuals. The MFC unit size investigation allows for a better evaluation of the inactivation efficacy and therefore a better design for the implementation. This provides a wider investigation into both Gram-positive (*Staphylococcus aureus*) and Gram-negative (*Salmonella enteritidis* serovar *Typhimurium* and *P. aeruginosa*) species.

## Materials and methods

### MFC reactor construction and operation

Three different trials were conducted, which employed two different sizes of MFCs; in the first trial (Trial 1), smaller MFCs were used with only *S. typhimurium* tested as the target pathogen, whereas in the second trial (Trial 2), larger MFCs were tested with all three pathogens as the target species; to separate the pathogenic species, the experiments with *S. aureus* were called “Trial 3”, but were conducted with the same larger MFCs of Trial 2.

The smaller MFCs in Trial 1 were built from earthenware ceramic membrane and supplied with carbon veil anode and carbon-painted cathode. Their design has been described in detail previously [27]. The internal volume of each empty MFC was 11.4 mL. The MFC setup consisted of two MFC cascades and was identical as previously described [20]. The open circuit cascade consisted of six individual MFCs and the closed circuit cascade consisted of nine MFCs. The external load connected to the MFCs under the closed circuit conditions was 250 Ω, and this had been determined from previous polarisation experiments as being close to the optimum for maximum power transfer. Human urine was supplied to each cascade at a constant flow rate of 400 mL/day, resulting in hydraulic retention time of 0.3 h per each individual MFC.

**Fig. 1:**
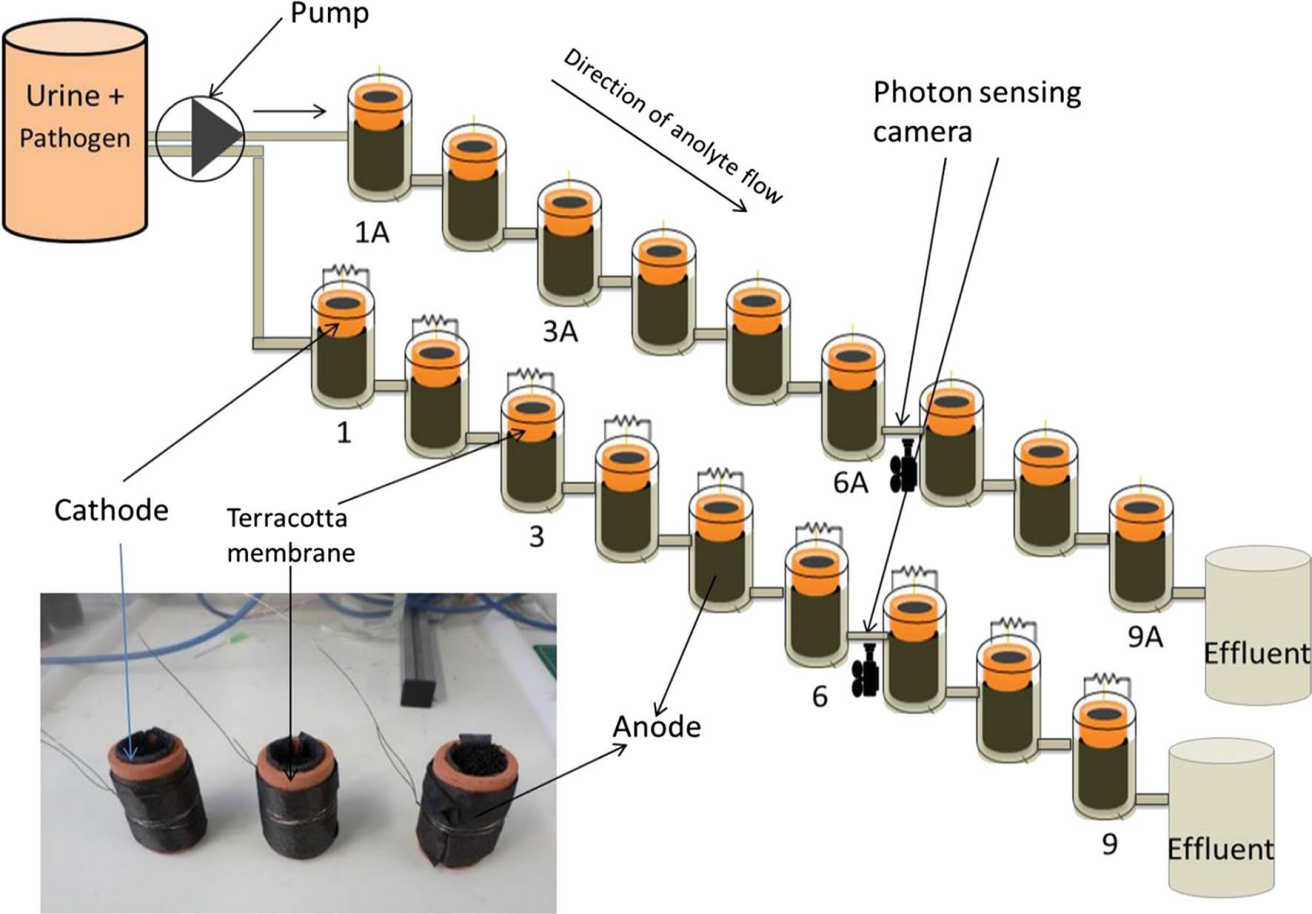
Experimental setup showing the two cascades of nine MFCs for Trials 2 and 3; *MFC 1–9* closed circuit (with 100Ω of external resistance applied), *MFC 1A–9A* open circuit (without any external resistance). Trial 1 was identical but with six MFCs in the open circuit cascade. A dark cabinet was used for real-time bioluminescence measurements using photon detectors. Inset: terracotta MFCs. MFC numbers 1, 3, 6, 9 represent sampling points for bench top luminometer readings and viable counts

For Trial 2, MFCs were assembled using terracotta ceramic cylinders sealed at one end (Orwell Aquatics, UK) with the following dimensions: length 10 cm, outside diameter 2.9 cm, inside diameter 2.1 cm, and wall thickness 4 mm. The anode electrode was made of carbon veil (carbon loading 30 mg/cm^2^) with a macro-surface area of 300 cm^2^, which was folded and wrapped around the terracotta tube with the use of nickel chromium (Ni–Cr) wire for current collection. The cathode was made of activated carbon [30% wet proofed with polytetrafluoroethylene (PTFE)] as previously described [7]. The 30 cm^2^ activated carbon-coated cathode was inserted into the cylinder, held against the ceramic wall by inserting a rubber sponge and connected via stainless steel crocodile clip. The MFC was placed in a plastic container (~ 60 mL working volume) where the outer anode surface was fully immersed into the anolyte (urine). The multi-channel data logger 34972a (Farnell, UK) and the electrical load were connected to the Ni–Cr wire, which was wrapped around the electrode to collect the electrons from its surface. The setup included two individual MFC cascades, consisting of nine MFCs each operated under closed circuit, with an external resistance of 100 Ω (obtained from polarisation experiment - see Fig. S2) applied (1–9) and another nine used as controls (1A–9A), which were operated under open circuit conditions, with no external resistance applied (no power generation). The MFCs within each cascade were connected as shown in Fig. 1, but were separated by physical air gaps which allowed inter-unit isolation, to avoid conductive bridging.

The MFC anodes had already been established over a 120-day period, as part of a previous experiment, which was originally inoculated with activated sewage sludge (Wessex Water Scientific Laboratory, Saltford, UK). The influent consisted of neat human urine donated by adult volunteers with pH ranging from 6.45 to 7.1. The MFC cascades were fed with collected urine at a flow rate of 400 mL/day, which resulted in a hydraulic retention time of approximately 3.5 h. External load applied to the “closed circuit” MFC cascade was 100 Ω, determined from polarisation experiments, carried out previously on identical MFCs.

### Data capture and power output

The potential of MFCs was recorded in volts (V) against time by a multi-channel Agilent data logging device. Recorded data were processed and analysed using the GraphPad Prism version 5.01 software package (GraphPad, San Diego, USA).

The current (*I*) in amperes (A) was calculated using Ohm’s law, *I* = *V/R*, where *V* is the measured voltage in volts (V) and *R* is the known value of the external load resistor in ohms. Power (*P*) in watts (W) was calculated by multiplying voltage with current, *P *=* I* × *V*. Power density was calculated in terms of the electrode surface area, *P*_Density_ = *P*/*v*, where *v* is the volume of the anodic chamber (mL) as previously described [14]. A Hanna 8424 pH meter (Hanna, UK) was used for taking the pH measurements.

#### Pathogen inactivation trials

The pathogens used in both trials were obtained from the collection of the University of the West of England. All strains carried the pBBR1MCS-2 plasmid derivative containing the luxCDABE operon of *Photorhabdus luminescens, w*hich allowed in situ, real-time monitoring of the presence of pathogens and rate of metabolism. To maintain the plasmids within pathogen cells, urine was supplied with gentamicin (10 mg/L) (*S. typhimurium* and *P. aeruginosa*) and erythromycin (*S. aureus*). The urine–pathogen mix was made up and pumped into the cascade at the start of the experiment. A piece of anode material (2 cm × 2 cm) was attached to the anode at the start of the experiment, to evaluate the attachment of the test organisms to the anode materials and their tendency of forming biofilms on the anode. At the end of the test experiment (24 h later), this piece of anode material was carefully removed using forceps and placed in 1 mL PBS buffer (pH 7). This was then vortexed briefly, serially diluted and plated on selective media.

To investigate the survival of pathogenic species in neat urine, 1 mL of urine was collected from the inlet tank and analysed for viable counts at the end of the experiment. Viable counts (colony-forming units/mL) from *T*_0_ (before introduction into the MFCs), *T*_x_ (urine–pathogen mix not introduced into the cascades, but set aside till the end of the experimental test) and from sampling points 1, 3, 6, 9 along the MFC cascades were performed by plating serially diluted replicates of the collected samples on selective nutrient agar and incubated at 37 °C for 16–24 h. Log-fold reduction of colony-forming units and bioluminescence were calculated using the following formula:

$${\text{LR}}\, = \,{ \log }\left( {A/B} \right),$$

where *A* is the number of viable microorganisms or bioluminescence intensity of the urine–pathogen mix before introduction into the cascades and *B* is the number of viable microorganisms or bioluminescence intensity at various sampling points (effluents of MFC 1, 3, 6, 9), as previously described [12]. Bioluminescence measurement of the collected 1 mL samples was conducted using a single-tube FB12 Luminometer (Berthold Detection Systems, Germany) in relative light units (RLU). The standard deviation (SD) was calculated using the log reduction values obtained from the replicated analysis. A two-way analysis of variance (ANOVA), with 95% confidence interval was conducted using GraphPad Prism Version 5.01 to test differences between the closed circuit and open circuit cascades.

In all cases, the model pathogenic strains were added to the MFCs with established electroactive biofilm generating stable electricity.

### Trial 1

In the first trial, *S. typhimurium* strain was introduced into the small-scale, MFCs in the same manner as described previously in detail for *S. enteritidis* [12]. In brief, two individual MFC cascades: open circuit and closed circuit cascade were used in parallel to treat human urine, into which *S. typhimurium* was introduced (at an initial density of 10^5^ CFU/mL). Overall, the study and experimental setup in Trial 1 was constructed in a similar manner to Trial 2 (as shown in Fig. 4), where the same *S. typhimurium* strain was also tested. The aim of using two different experimental setups was to compare the inactivation efficacy from two different MFC sizes.

### Trial 2

In Trial 2, two identical Hamamatsu photon-detecting cameras (Hamamatsu Photonics, K.K., Japan) were purchased and calibrated to the same settings. Both photon detectors were inserted after MFC #6 of the open and closed circuit MFC cascades. In Trial 2, *Salmonella typhimurium* and *P. aeruginosa* were grown in nutrient broth (100 mL each) until they reached the density of 10^13^ CFU/mL, harvested by centrifugation, re-suspended in 1 L of influent urine (with gentamycin 10 mg/L) and pumped through the cascades at 17 mL/h. The urine/pathogen mix (density of 10^12^ CFU/mL) was stirred periodically to prevent sedimentation of pathogens.

The photon sensors for Trials 2 and 3 (see Fig. 4) were placed after MFCs #6 in both closed and open circuit cascades based on observations from preliminary tests which indicated that substantial pathogen decline was recorded after MFC #6 (Fig. S1). The use of two photon cameras made it possible to measure pathogen presence (bioluminescence) at two different but equivalent locations simultaneously. At the end of the trial, samples were collected from MFCs #1, 3, 6, 9 (outflows) for offline luminescence and viable counts analyses. The real-time and offline luminometer measurement was carried out on two pathogenic bacteria (*S. typhimurium* and *P. aeruginosa*) introduced into the cascades in Trial 2.

### Trial 3

Trial 3 was conducted in a similar manner to Trial 2. However, in this case the bioluminescent pathogen *S. aureus* was used as a model strain, with the difference that it was initially grown in 150 mL nutrient broth until a density of 10^10^ CFU/mL, centrifuged (11,000×*g* at 4 °C for 10 m) and re-suspended into 1 L of urine containing erythromycin (10 mg/L) giving a final concentration of 10^9^. The cascade position for the two photon sensors was the same as in Trial 2. At the end of the trial, samples were collected from MFCs #1, 3, 6, 9 (outflows) for offline luminescence and viable counts and analysed in triplicate.

The present MFC configuration, using small terracotta ceramic cylinders (Fig. 1) was recently demonstrated to be an efficient ceramic material for power generation. For this study, we evaluated the fate of different pathogens within the anodic chamber of terracotta ceramic material covering a wider range of species (Gram-positive and Gram-negative, as well facultative anaerobic bacteria).

## Results and discussion

### MFC performance and power generation

**Fig. 2:**
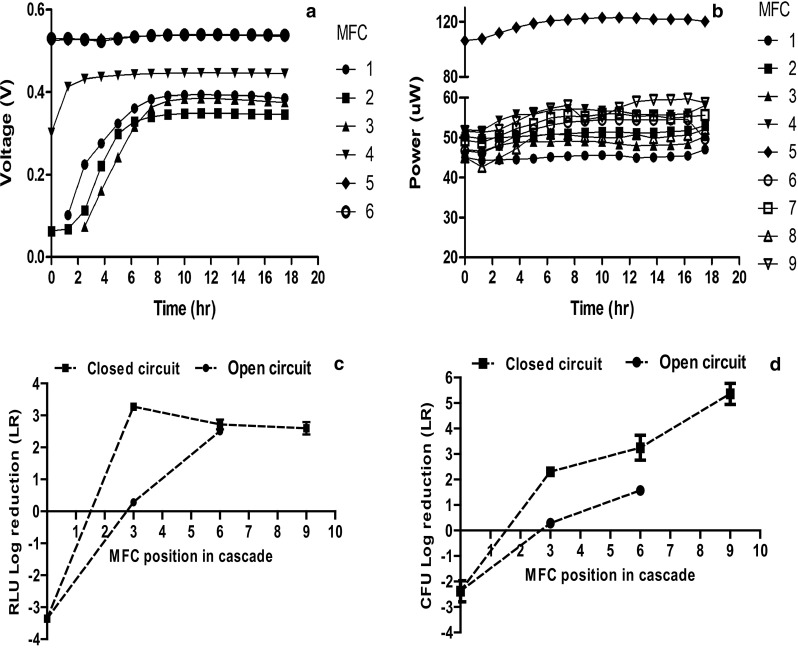
Results of Trial 1, where low-power, small-scale MFCs were used to test the fate of *S. typhimurium* strain: **a** Potential of the open circuit cascade. **b** Power output of closed circuit cascade. **c** Results of the log-fold reduction in bioluminescence and **d** colony-forming units (CFU)

The performance in terms of absolute power recorded during Trial 1 was significantly lower than for the two other trials carried out using the larger MFCs. The average open circuit voltage observed for the OC cascade was of 401 ± 136 mV (Fig. 2a), whilst the average power at the end of the trial was 59 ± 22 µW (Fig. 2b). This was at least one order of magnitude lower than the levels of power produced in Trial 2 and Trial 3. This difference may have been due to the size difference amongst several other factors; however, it is important to note that the power density as well as inactivation efficacy was higher for the smaller MFCs of this Trial 1 (see further below).

**Fig. 3:**
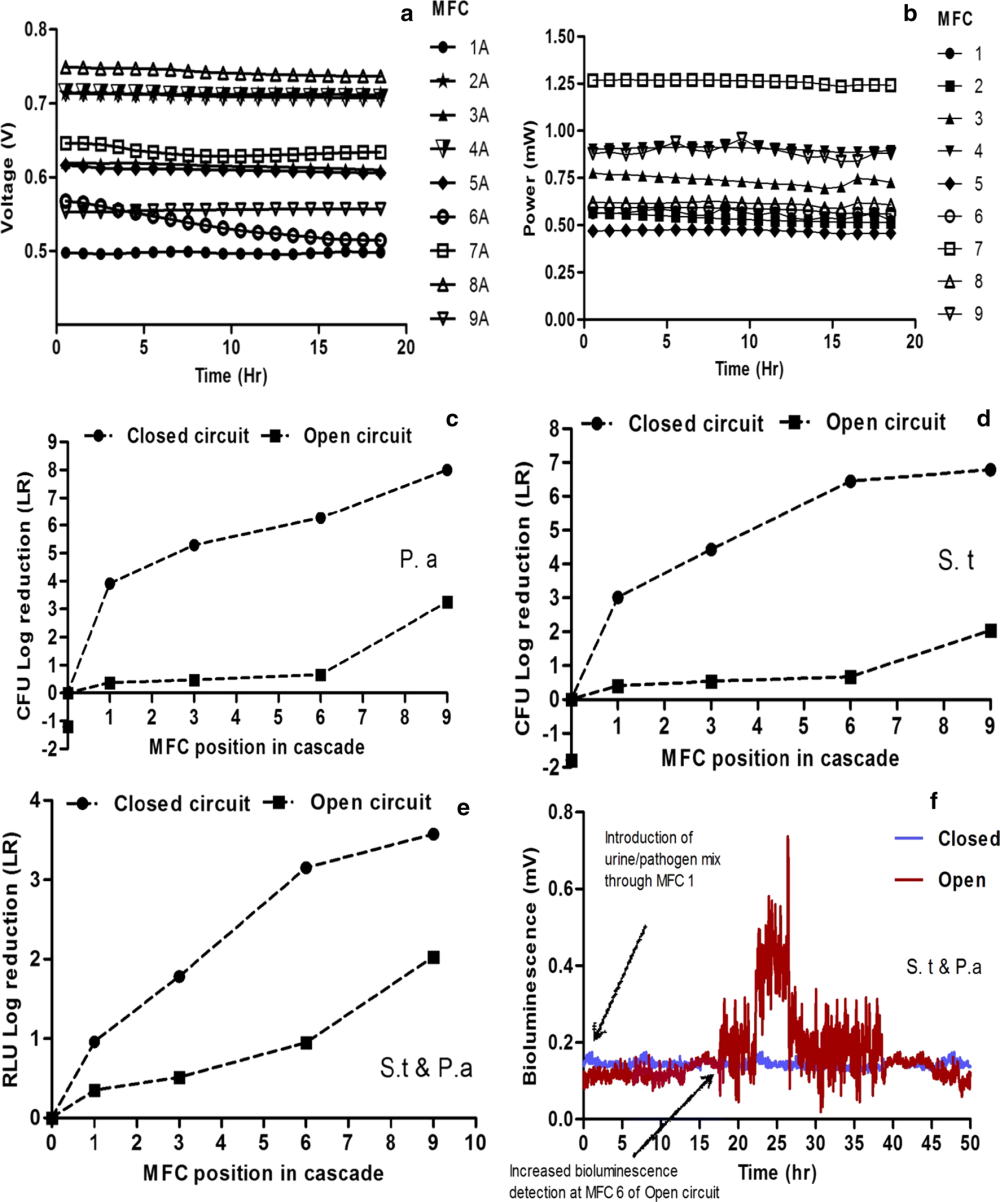
Results of Trial 2: electrical output levels from **a** open and **b** closed circuit cascades; **c** log-fold reduction of viable counts of *Pseudomonas aeruginosa* introduced to the closed and open circuit cascades; **d** log-fold reduction of viable counts of *Salmonella typhimurium* introduced to the closed and open circuit cascades; **e** log-fold reduction of luminometer measurements from the cascades inoculated with pathogenic *S. typhimurium* and *P. aeruginosa*. **f** Real-time bioluminescence monitoring of both *S. typhimurium* and *P. aeruginosa* treated in open and closed MFC cascades. Photon detector was located after MFC #6 in both cascades. LR data are represented by the average of three replicates ± standard deviation

**Fig. 4:**
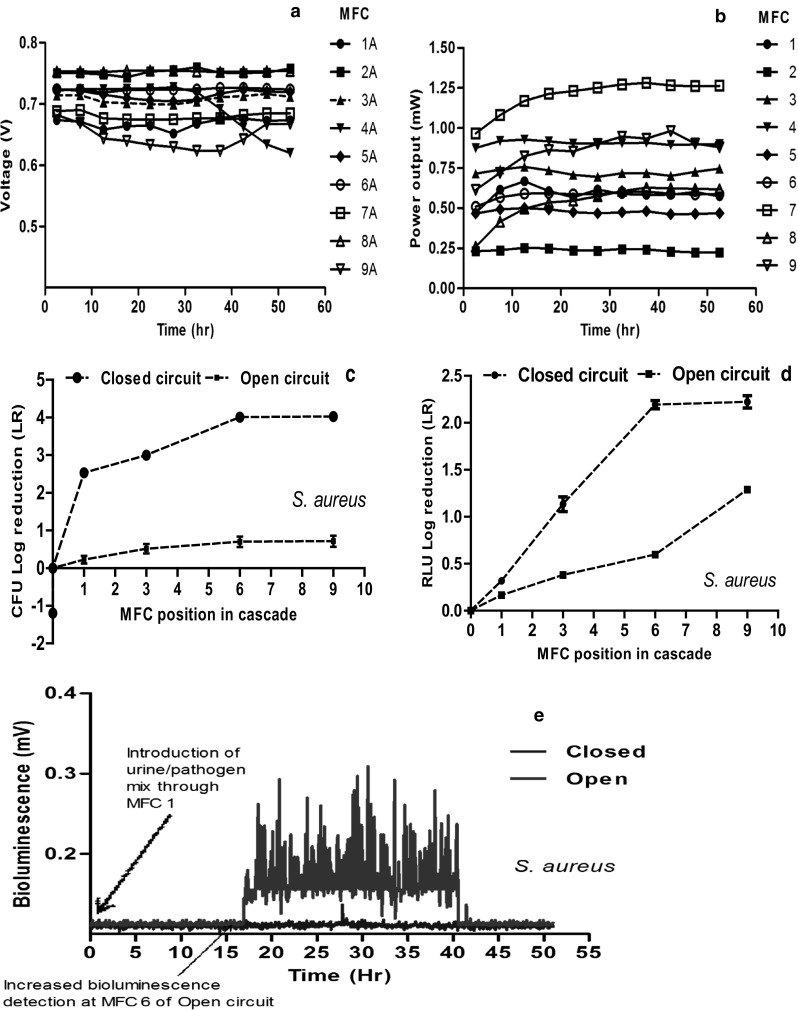
Results of Trial 3. Power performance of **a** open and **b** closed circuit cascades; **c** log-fold reduction in viable counts (CFU/mL) of pathogenic *S. aureus*. **d** Log-fold reduction of bioluminescence along the cascades; **e** real-time bioluminescence monitoring of pathogenic *S. aureus.* Detector located after MFC #6 in both cascades. LR data are represented by the average of three replicates ± standard deviation

The MFC performance observed during Trial 2 and Trial 3 resulted in different levels of power output from otherwise identical MFCs within the cascades. As the MFCs were only connected hydraulically, each MFC performed as an independent, electrically isolated unit, and was not affected by the performance of other MFCs via fluidic electrical conductance. Furthermore, the results of power generation suggest that the performance of the MFC units was largely independent of their position in the cascade. Power output in the closed circuit cascade of the trials 2 and 3 remained stable, with some of the MFCs generating nearly or over 1 mW, whilst other MFCs were producing power below 0.5 mW. This disparity might have resulted from the manual preparation of these MFCs, the contact between the electrodes (esp. the cathode) and the ceramic membrane as well as the differential colonisation of the anode electrode by different electroactive bacteria. Nevertheless, the stability observed in power output from the individual MFCs suggests that metabolic steady states had been achieved, since this parameter is directly proportional to power generation. The average voltage output of the open circuit cascade was 630 ± 079 mV (Fig. 3a), while the average power output of the closed circuit MFC cascade from Trial 2 reached 763 ± 191 µW (Fig. 3b). For Trial 3, the average voltage output of the open circuit cascade was 703 ± 078 mV (Fig. 4a), while the average power output of the closed circuit MFC cascade reached 718 ± 123 µW (Fig. 4b).

### Pathogen inactivation analysis

#### Trial 1

The pathogenic *S. typhimurium* introduced into the small-scale MFCs was efficiently removed despite the relatively low-power output. The average log reduction of CFU observed for the closed circuit cascade was equal to 5.22 ± 0.26 for MFC9 and 3.09 ± 0.29 for MFC6 (over 18 h of operation) and outperformed the open circuit cascade composed of six MFCs with LR equal to 1.57 ± 0.06, which showed that the CFU–LR of the closed circuit was significantly higher than that of the open circuit cascade when similar MFCs were compared (*P *< 0.05). Similarly, the MFC environment had an inhibitory effect on the metabolic activity of bioluminescent *S. typhimurium* revealing 4.21 ± 0.01 RLU–LR values (MFC3) and 3.98 ± 0.02 (MFC6) against 3.13 ± 0.02 observed in open circuit control (Fig. 2c, d). The reduction in pathogen numbers corresponds to an increase in pH from 6.97 in the influent to 9.61 within the reactors. Similarly, there was a significant reduction in oxidation–reduction potential from − 65 in the influent to − 450 mV in Trial 1. These changes have probably resulted in a hostile environment for the alien pathogen, which in turn resulted in the observed reduction.

The results indicated that small-scale MFCs were able to induce disinfection and efficiently deactivate pathogenic *S. typhimurium* cells. As with the absolute values in power output, the non-normalised LR–CFU values were also found to be lower in Trial 1, when compared to the results obtained in Trial 2 and Trial 3, where MFCs produced at least one order of magnitude higher than the previous power output (see Table 1).

**Table 1:** Summary of results obtained from the three individual trials of pathogen inactivation in urine-powered MFC cascade

Trial	MFC no.	Closed circuit			Open circuit			Δ CFU–LR in CC and OC cascades
		Power (µW)	Luminometer (RLU–LR)	Viable counts (CFU–LR)	Voltage (mV)	Luminometer (RLU–LR)	Viable counts (CFU–LR)	△ CC vs OC
#1	6	62 ± 22	3.98 ± 0.02	**S.t:** 3.09 ± 0.29	401 ± 136	3.13 ± 0.02	**S.t:** 1.57 ± 0.06	**S.t:** 2.02
	9	59 ± 22	4.21 ± 0.01	**S.t:** 5.22 ± 0.26	na	na	na	na
#2	6	634 ± 173	3.15 ± 0.02	**S.t:** 6.45 ± 0.06	612 ± 085	0.95 ± 0.02	**S.t:** 0.67± 0.09	**S.t:** 5.78
	9	763 ± 191	3.80 ± 0.11	**S.t:** 6.91 ± 0.12	630 ± 079	2.02 ± 0.01	**S.t:** 2.04 ± 0.1	**S.t:** 4.87
	9	763 ± 191	3.80 ± 0.11	**P.a:** 7.79 ± 0.11	630 ± 079	2.02 ± 0.01	**P.a:** 3.25 ± 01	**P.a:** 4.54
#3	9	718 ± 123	2.01 ± 0.11	**S.a:** 4.01 ± 0.10	703 ± 078	1.41 ± 0.02	**S.a:** 0.71 ± 0.1	**S.a:** 3.30

Results show average of power output from MFCs in the cascade and average of triplicate LR analysis of influent and effluent from closed and open circuit cascades ± SD

*RLU* relative light unit, *CFU* colony-forming unit, *LR* log-fold reduction, *S.t S. typhimurium, P.a P. aeruginosa, S.a S. aureus*, *OC* open circuit, *CC* closed circuit, *na* not available

#### Trial 2

To evaluate the viability of the pathogenic bacteria introduced into the MFC cascade, offline luminometer analysis was carried out to measure their bioluminescence at different points in the cascade system. The results showed significantly higher log-fold reduction in bioluminescence of the samples taken from the closed circuit (*P *< 0.05), power-generating MFC cascade than from the open circuit cascade. Specifically, there was a 3.8 ± 0.06 log-fold reduction of bioluminescence for the closed circuit cascade compared to 2.01 ± 0.01 log reduction recorded for the open circuit cascade (Fig. 3e). These results indicate that the characteristics of the power-generating MFC cascade were more inhibitory to the exogenous pathogenic organisms than the open circuit cascade resulting in greater reduction or suppression of the metabolic activities of the pathogens.

Our previous research has shown a direct correlation between bioluminescence from pathogenic species (whether in real time or offline), and viability/metabolic rate [8, 12]. Therefore, these results suggest that the power generation process was able to bring about significant reduction in the viability and metabolic rate of the target pathogens.

To further test the findings from the luminometer analysis, effluent samples collected at different sampling points within the MFC cascades were inoculated onto selective agar media for viable counts and analysed in terms of colony-forming units (CFUs). The results showed significantly higher log-fold reduction (LR) of CFUs of the samples taken from the closed circuit cascade than from the open circuit cascade (*P *< 0.05). For the power-generating closed circuit MFC cascade, CFU–LR of 7.79 ± 0.01 and 6.91 ± 0.04 were observed for *P. aeruginosa* and *S. typhimurium,* respectively, whilst LR of 3.25 ± 0.05 and 2.04 ± 0.13 were recorded in the open circuit cascade, respectively, (Fig. 3c, d), resulting in a difference of almost fivefold in logarithmic terms in both cases (Table 1). Two-way analysis of variance (ANOVA) at 95% confidence interval showed the observed difference between the closed and open circuit cascade is statistically significant in both cases (*P *< 0.05).These results were in agreement with the data obtained from luminometer analysis.

Furthermore, the results from the real-time bioluminescence monitoring revealed strong signals from the open circuit cascade, while only a low signal was recorded for the closed circuit cascade (Fig. 3f), which corroborate with the previous luminometer and viable count results. This indicates that many of the *S. typhimurium* and *P. aeruginosa* cells introduced into the open circuit cascade remained largely viable and active, while those in the close circuit cascade were significantly inhibited and inactivated. The results from CFU and RLU (both online and offline) measurements indicated, that open circuit MFCs were inefficient in disinfection of both model organisms. In contrast, survival and activity of both pathogenic species in closed circuit MFCs were negligible.

#### Trial 3

In Trial 3, we assessed the fate of *S. aureus* in urine-fed MFCs cascades operated in a continuous flow. The results of offline bioluminescence measurements conducted on the effluents of selected MFCs revealed a significantly greater reduction (*P* < 0.05) in the bioluminescence across the closed circuit cascade than the open circuit cascade. The LR value reported for open circuit MFCs was 1.41 ± 0.2, while for the closed circuit cascade they reached 2.01 ± 0.1. This reduction is an indication of the ability of the power-generating MFC to deactivate *S. aureus* cells in addition to Gram-negative pathogens when introduced into the cascades (Fig. 4d).

The result of bioluminescence assay was further corroborated by the viable counts measurements of the effluents, which also showed significantly greater reduction (in CFU) of the power-generating cascade (*P *< 0.05) when compared to the open circuit cascade. Specifically, a CFU–LR of 4.01 ± 0.11 was recorded in the closed circuit cascade in contrast to only 0.70 ± 0.15 for the open circuit cascade (Fig. 4c). Since other parameters of the MFCs were kept the same, the results suggest that the power-generating process of the closed circuit MFCs was the major mechanism facilitating the inactivation of the pathogenic *S. aureus.* The results of real-time bioluminescence monitoring of the viability of the pathogenic *S. aureus* also showed that after MFC #6, the detection of the pathogens was negligible in the closed circuit cascade, while strong bioluminescence signals were recorded for a period of 25 h in the open circuit cascade (Fig. 4e), an indication that while the *S. aureus* introduced into both cascades were alive and active in the open circuit cascade, they were inhibited and inactivated in the power-generating closed circuit cascade. Table 1 provides a summary of both power and biological parameters obtained from the three trials.

The results observed in our current study (Trial 1, 2 and 3) showed that the introduction of large quantities of real pathogens into the MFC cascade had no negative impact on power generation and by extension, on the electroactive biofilm community. They also highlight the unique abilities of closed circuit MFCs to not only generate usable power, but also achieve inactivation of pathogenic organisms introduced into the cascades.

The three pathogenic organisms tested in the current study have been reported to form biofilms on different types of substrata [2, 5, 35], which could result in their retention within the anode of microbial fuel cells, thereby giving false reduction levels. In the current study, we found no indication of pathogen attachment to the anode either through the real-time power output monitoring or through the analysis of the anodic biofilm. Only negligible quantities of pathogen cells were detected (Table 2).

**Table 2:** Distribution of remnant pathogens (*P. aeruginosa* and *S. typhimurium*) in urine-fed MFC cascades 24 h after pathogen test in attached piece of anode (4 cm^2^) and within the anolyte

	Closed circuit	Closed circuit	Open circuit	Open circuit
	*P. aureginosa* (CFU/mL)	*S. typhimurium* (CFU/mL)	*P. aureginosa* (CFU/mL)	*S. typhimurium* (CFU/mL)
*Attached*				
T1	0	0	170	64
T3	86	68	366	88
T6	77	97	567	104
T9	100	66	840	98
*Planktonic*				
T1	129	0	132	80
T3	213	5	210	275
T6	353	34	550	434
T9	344	33	734	373

*Planktonic* number in the liquid within the anode, *Attached* those found on the piece of anode material attached to the anode

Our previous study involving in situ pathogen inactivation in MFC cascades using *S. enteritidis* [8] has also shown that the pathogenic bacterial cells were not incorporated into the anodic biofilm of either closed or open circuit cascade. As such, it is unlikely that the pathogens form a biofilm on the anode in the current study. However, evaluation of the pathogens viability in the inlet tank showed an increase in the number of pathogens, which suggests that keeping the pathogens in urine for up to 35 h, does not inhibit their growth during the duration of the experiment.

Previous research has shown that most of the microorganisms within the anodic chamber whether planktonic or attached potentially contribute to the power-generating process. These microorganisms might be electroactive or not, but contribute to the electricity generation as a result of their normal metabolic activities where they could provide suitable substrates to electrogenic bacterial community thereby aiding the power generation process [13]. Research has also shown that even some pathogenic organisms such as *P. aeruginosa* have been isolated from the electrogenic community within the anodic chamber of MFCs [29]. More so, introduction of *P. aeruginosa* for instance, from an aerobic to anaerobic environment as is the case in the current study has the potential to enhance its electrogenic abilities as a result of improved anode biofilm formation by the organisms [38]. However, the addition of large numbers of pathogenic organisms into the anodic chamber in the current study did not lead to sudden increase in power output suggesting that their contribution to electricity generation, if any, is negligible. In all cases, the pathogenic organisms were introduced in already colonised anodes in well-established, power-generating MFCs. It is worth investigating whether pathogens can contribute to the generation of power when they form part of the inoculum (i.e., inoculating a fresh cascade with contaminated urine/sludge) and this will form a part of our near term experimental work.

### Mechanisms of disinfection in urine-powered MFC cascades’ system

Several factors have been proposed to be responsible for the suppression of growth of exogenous organisms introduced into power-generating MFC cascades, and one of these is the high pH (> 9.5) recorded in urine-powered MFCs [12], which is inhibitory to bacterial growth. However, observation from the current study showed a negligible difference in pH recorded between closed and open circuit cascades. The pH of the open circuit cascade effluent (9.66) was in fact slightly higher than that of the closed circuit cascade (9.50), where significant pathogen inactivation was observed. Therefore, although high pH might have inhibited the growth of the pathogens, it was not shown to be the major factor in the pathogen inactivation processes in the current study.

One of the major differences between the open and closed circuit cascade was the sequential application of external resistance from 1500 Ω (at the start) to 100 Ω at the later stages of the MFC operation. External resistance affected not just the anode potential but also the development of the anodic biofilm and as such also affected current generation [17]. The development, diversity and distribution of anodic biofilm are affected by the external resistance, because of its influence on the degree of adaptation of the electroactive communities. Higher external resistance has been shown to result in better enrichment of the anode as well as more diverse anodic biofilm community than low external resistance [17, 33]. Although these reports highlight the significance of external resistance in the selection, adaptation and distribution of various anodic biofilm communities, they did not provide information on the impact of external resistance on exogenous bacteria, which were not part of the already developed biofilm.

Intra- and interspecific interactions including competitions and cooperation are important factors that could have influenced pathogen survivability within the anode of the MFCs. Research has shown that *P. aeruginosa*, for instance, is able to thrive in almost all environments because of its ability to interact with other bacteria within the environment. One of the unique qualities of *P. aeruginosa* is the ability to release chemical signals with which to either cooperate with other bacteria to enhance its survival or to antagonise other bacteria in competition for the limited carbon source [19, 34]. As such, competition and other forms of inhibitory interactions between exogenous bacteria and the already established attached and planktonic resident anodic communities for space and nutrients might also have resulted in the decline of the exogenous bacteria. However, these interactions would occur within both closed and open circuit cascades.

The electricity generation process, which is the biologically driven electrochemical reaction of the closed circuit cascades leads to reduced oxidation–reduction potential (ORP). Although this mechanism requires a dedicated and detailed study, we believe that ORP is the major contributing factor for pathogen inactivation. Studies on the impact of changes in redox potential levels indicated that a decrease in redox potential under stressful conditions results in growth cessation in a number of common bacteria including *E. coli* and *B. subtilis* [30, 31]. Furthermore, it has been shown that the electrode potential has a critical impact on the survival and development of bacteria within the MFCs [28]. The sharp decline in ORP in the current trials from − 50 in the influent to − 125 in the effluent in trials 2, 3 could have led to stressful conditions, which would inhibit the growth of the pathogens.

This is evidenced by the significantly higher pathogen log-fold reduction recorded in closed circuit cascades in Trial 2 and Trial 3, when compared to Trial 1, where the same *S. typhimurium* strain was used in our previous study, but in smaller scale, lower power MFCs. The absolute power in the present work was tenfold higher in Trial 2 and 3, than in Trial 1 or indeed in the previous study [12], which may account for the higher log-fold reduction levels (6.91 vs 5.22) and higher Δ log-fold reduction between closed and open circuit cascades (4.87 vs 3.65 for MFC9 and vs 2.02 for MFC6), respectively. However, by calculating the inactivation efficacy as “density”, i.e., normalised to the MFC size (volumetric), then the outcome is different. The MFCs in Trial 1 had an anodic volume of 11.4 mL, which (taking into account the 18.7 mL/h flow rate) resulted in a dilution rate of 1.64/h and a cascade HRT of 5.94 h. This means that the inactivation rate was 0. 88 LR/h (MFC9) and 0.51 LR/h for MFC6. Using the same logic, the larger MFCs in Trials 2 and 3 achieved an inactivation rate of 0.22LR/h or 0.33 LR/h for MFC 6 (for *S. typhimurium*) and 0.24LR/h and 0.12LR/h for *P. aeruginosa* and *S. aureus*, respectively. The inactivation rate for the smaller MFCs was therefore 4 × faster than that of the larger MFCs, and this may be attributed to the MFC design, volume, fluid dynamics as well as power performance.

The pathogenic organisms tested in the current study belong to different phyla of bacteria, including representatives of Gram +ve and −ve. It is possible that similar faecal–oral pathogens such as *Campylobacter jejuni*, pathogenic *E. coli*, *Plesiomonas shigelloides, Salmonella typhi, Shigella* spp., *Yersinia* spp. and *Vibrio cholera* [25] could be potentially inactivated by the same conditions as well as the external resistance within a power-generating anodic chamber of MFC cascade. However, specific tests involving some more virulent pathogens endemic in the developing countries are underway to evaluate their fate within the anodic chamber of power-generating MFCs.

### Implications of pathogen inactivation in MFCs cascade systems

Waterborne pathogens are introduced into the drinking-water supplies mainly as a result of faecal contamination and improper sanitation and waste management. Some pathogens such as *Salmonella* spp., *P. aeruginosa* and *S. aureus* cause several kinds of infection in humans including diarrhoea, septicaemia, meningitis, endocarditis, osteomyelitis, pneumonia and typhoid fever which are still regular occurrences within communities in developing countries [10]. While a number of approaches have been utilised to enhance the ability of wastewater treatment plants to remove pathogenic organisms during water treatments, including the use of filters [31, 40], cases of large numbers of *Salmonella* spp. have been reported in wastewater effluents [20]. Previous studies have shown that the secondary and tertiary treatment could only produce 2–3 log-fold reduction in the number of pathogenic organisms including faecal coliforms and *E. coli* [9]. This is currently not sufficient to achieve the established discharge limits, especially for wastewater reuse in irrigation, whilst the use of chlorine and its derivatives, which appear effective, raise concerns about dangerous by-products [40]. The results of the current study highlight the susceptibility of the three tested pathogens to the treatment within power-generating MFC cascades. The MFC technology therefore offers a promising, low cost solution to the challenge of sanitation, and power generation, and could become a vital technology for the disinfection of liquid waste in the future, especially with continuous improvement of the power generation potentials of the technology.

## Conclusions

MFC technology is becoming a strong contender in the bioenergy sector, as more of its applications are discovered. The current study highlights the potential of a power-generating, urine-fed MFC cascade effecting significant reduction in the numbers of exogenous organisms introduced into the system. This is being achieved as a result of the unique conditions within a power-generating anodic chamber of an MFC, which creates a stressful environment for the exogenous pathogenic organisms. MFC cascade systems therefore offer a unique solution that could prove useful for off-grid, low cost, sanitation and power generation in poor, remote areas around the world, places where the technology is thought to have ‘comparative advantage’ as a result of energy needs and suitable weather conditions.

## Electronic supplementary material

Below is the link to the electronic supplementary material.

Supplementary material 1 (DOCX 122 kb)
