# Supplementary material for: Fate of three bioluminescent pathogenic bacteria fed through a cascade of urine microbial fuel cells
Source: J Ind Microbiol Biotechnol. 2019 Feb 22;46(5):587–99. doi: 10.1007/s10295-019-02153-x (PMC6510811; doi:10.1007/s10295-019-02153-x)
Supplement: Supplementary file 1 — Supplementary material 1 (DOCX 122 kb) [file 10295_2019_2153_MOESM1_ESM.docx]

Preliminary pathogen test, using pathogenic, bioluminescent *E. coli*.

Fig. S1. CFU Log reduction in E coli introduced into working closed and open circuit MFC cascades (preliminary results). The results shows that significant reduction was achieved after MFC 6; as a result, the photon sensor (camera) was placed after MFC 6 in subsequent tests.

Fig S2. Polarization curve for the closed circuit microbial fuel cell cascade with an established anode biofilm.
